# Primary Graviceptive System and Astasia: A Case Report and Literature Review

**DOI:** 10.3390/brainsci13101371

**Published:** 2023-09-26

**Authors:** Ko-Ting Chen, Sheng-Yao Huang, Yi-Jye Chen, Ying-Yun Chen

**Affiliations:** 1 Department of Neurosurgery, Chang Gung Memorial Hospital at Linkou, Taoyuan 333, Taiwan; 2 Neuroscience Research Center, Chang Gung Memorial Hospital at Linkou, Taoyuan 333, Taiwan; 3 School of Medicine, Chang Gung University, Taoyuan 333, Taiwan; 4 Molecular Medicine Research Center, Chang Gung University, Taoyuan 333, Taiwan; vic.huang1973@gmail.com; 5 Department of Ophthalmology, China Medical University Hospital, Taichung 402, Taiwan; jessica1293hsnu@gmail.com; 6 Department of Neurosurgery, Chang Gung Memorial Hospital at Keelung, Keelung 204, Taiwan; ruby3816@gmail.com

**Keywords:** astasia, graviceptive system, subjective visual vertical, subjective posture vertical, thalamo-cortical projections, vestibule-thalamic pathway

## Abstract

Astasia refers to the inability to maintain upright posture during standing, despite having full motor strength. Impairment of the vestibulocerebellar pathway, graviceptive system, and cingulate motor area have been proposed to be related to astasia. However, the responsible neural pathways remain unclear. We hypothesize that there is a common neural network behind astasia. To test the hypothesis, we reviewed all reported cases with astasia, including ours, and focused on the correlation between anatomical destruction and symptom presentation. A total of 26, including ours, non-psychogenic astasia patients were identified in the English literature. Seventy-three percent of them were associated with other neurologic symptoms and sixty-two percent of reported lesions were on the right side. Contralateral lateropulsion was very common, followed by retropulsion, when describing astasia. Infarction (54%) was the most reported cause. The thalamus (65%) was the most reported location. Infarctions were the fastest to recover (mean: 10.6 days), while lesions at the brainstem needed a longer time (mean: 61.6 days). By combining the character of lateropulsion in astasia and the presentation of an interrupted graviceptive system, we concluded that the primary graviceptive system may be the common neural network behind astasia. Future studies on astasia should focus on the pathological changes in the perception of verticality in the visual world and the body.

## 1. Introduction

Astasia refers to the inability to maintain an upright posture during standing, despite having full motor strength in four limbs [1]. There are no or minimal associated abnormalities in the neurological examination other than gait disturbance and truncal instability. Astasia is usually associated with abasia (inability to walk) in patients with central nervous diseases [2]. It was once recognized as a psychogenic or conversion disorder [3]; however, organic causes have engrossed this term in recent years. Several regions have been reported to be responsible for astasia, including the mesencephalon [4,5,6], thalamus [1,7,8], suprathalamic white matter [9], corpus callosum (CC) [10], cingulate gyrus [2,10,11], and supplementary motor (SMA) [12]. The causes are not limited to stroke [1,2,4,5,6,7,8,9,10,11,12,13] but also tumor [1], inflammatory disease [14], and hyper-perfusion [15]. However, the pathophysiology and neural pathways of astasia remain largely unknown.

A seminal paper by Masdeu [1] was the first to report on a series of thalamic astasia and had discussed in detail how to differentiate astasia from other causes of abnormal gait and station, such as cerebellar dysmetria, thalamic akinesia, sensory impairment, and hyperekplexia [1]. A bilateral projection of the vestibulocerebellar pathway from the fastigial nucleus was initially proposed to explain the affected pathway at the thalamus level and to explain the transient nature of thalamic astasia [1]. Since then, the majority of reported cases adopted the concept of lesions from the brainstem to cortical regions [4,5,6,10,12]. Apart from this, impairment of the graviceptive systems has been reported in patients with thalamic infarct at the centromedian nucleus [7] and posterolateral nucleus [8]. Lesions above the thalamus are distributed widely from the suprathalamic white matter [9] and corpus callosum (our case) to the cingulate gyrus [2,10,11] and SMA [12]; in these cases, a disturbance of the cingulate motor area (CMA) [2,11] and thalamo-cortical projections [9] were considered. In essence, a cerebello-vestibulo-thalamic-cortical pathway may be involved in generating astasia; however, the connections and neural networks between these speculated pathways remained unclear.

We described a patient with butterfly glioblastoma involving the body of CC and bilateral periventricular white matter who had isolated astasia-abasia as the initial presentation. After nearly a total removal of the tumor, he partially regained posture stability and balance, with the help of rehabilitation. We hypothesize that there is a common neural network among the vestibulocerebellar pathway, graviceptive pathway, and CMA behind the clinical presentation of astasia. To test the hypothesis, we reviewed all reported cases with astasia, including ours, in the English literature and focused on the correlation between anatomical destruction and symptom presentation. We propose that when the primary graviceptive system, a network to maintain the body’s upright posture, is interrupted, it results in astasia.

## 2. Materials and Methods

### 2.1. Case Illustration

#### 2.1.1. Preoperative Evaluation

**Figure 1:**
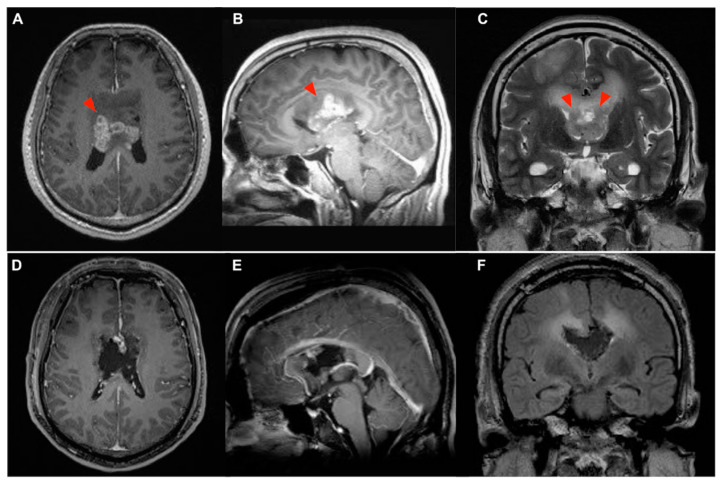
Pre- and postoperative brain MRIs. A large butterfly glioma arising from body of corpus callosum (CC) with heterogeneous enhancement ((**A**–**C**), red arrowheads) and T2 high signal extended bilaterally through interhemispheric fibers are shown. A separate non-enhanced lesion at right superior frontal gyrus was shown (**C**). Enlarged bilateral temporal horns (**C**) indicated an obstructive hydrocephalus induced by a bilateral occlusion of Foramen of Monro. The postoperative MRI showed a complete removal of contrast enhancing CC tumor (**D**–**F**) with a residual T2 high-signal area (**F**) and resolution of obstructive hydrocephalus (**F**).

A written informed consent was provided to the patient for this study. The data collection was approved by the Institutional Review Board to protect patient’s privacy. The 50-year-old man was a right-handed worker who presented with acute onset of gait unsteadiness progressively for 2 weeks. He was unable to stand straight on his own. When asked to sit up, he would grasp the side rails of the bed with both hands to pull himself up, without using axial muscles. When trying to walk under assistance, his base of gait was inconstant and unstable, and he easily fell toward either side or backward. However, his muscle power was grade 5/5 during the supine position and deep tendon reflex was normal, which did not suggest any peripheral neuropathy. Romberg’s test could not be performed, and the finger-nose-finger test showed no dysmetria. He was fully orientated but apathetic in appearance. There was no cranial nerve deficiency. The sensory function was intact. Other accompanying symptoms included dizziness, nausea, and short-memory decline. The above symptoms suggested a clinical diagnosis of isolated astasia-abasia. Magnetic resonance imaging (MRI) showed two separate lesions (Figure 1A–C), including a butterfly pattern intra-axial mass (6 cm) involving the body of CC, septum pellucidum, and body of fornix.

#### 2.1.2. Surgical Resection

The right frontal tumor (Figure 1C) was firstly removed after the somatosensory evoked a potential to confirm the primary motor cortex was behind the tumor. Then, a combined right transfrontal and bilateral interhemispheric approach was performed under neurophysiologic monitoring of the triggered motor evoked potential during the removal of the tumors with high signal intensity in the T2-WI. At the end of the resection, the bilateral lateral ventricle was entered, the fornix was invaded by the tumor which was partially restricted, and the third ventricle was under direct vision. The pathology of the right frontal lesion was a diffuse astrocytoma, IDH-mutant, WHO grade 2, and the tumor at the CC was an IDH-mutant glioblastoma. A postoperative MRI showed complete removal of the CC tumor (Figure 1D–F).

#### 2.1.3. Postoperative Clinical Course

The patient had a transient left-side weakness with a muscle power of grade 4 and left–central type facial palsy, which completely recovered within 2 weeks postoperatively. After intensive rehabilitation, a gradual, but significant, improvement in his sitting balance and posture control was noted. Six months later, although his daily life activities were still partially dependent (a Karnofsky performance score of 70), he was able to stand upright and walk under assistance, suggesting a partial recovery from astasia-abasia even after extensive removal of the body of CC.

### 2.2. Literature Review

**Figure 2:**
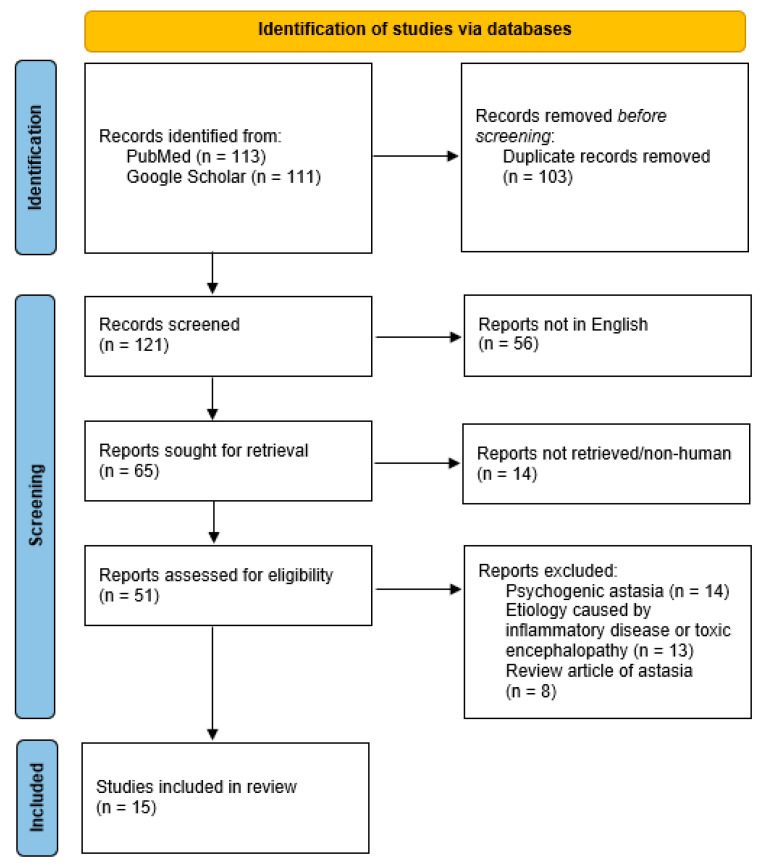
PRISMA 2020 flow diagram of systemic review for astasia case studies (n = 15).

As a template for the methodology, we utilized preferred reporting items and meta-analysis (PRISMA) guidelines for systematic reviews. In brief, the keyword “astasia” was used to search Pubmed and Google Scholar search engines. Topics related to psychogenic astasia, review articles, or cases caused by inflammatory diseases or toxic encephalitis were excluded. A total of 15 studies of organic astasia were included for a second review (Figure 2). From two reports in French [16,17], one report stated that the patient did not fall during an attack [18] (astasia unlikely) and another one with only an abstract available [19] were further excluded. Finally, clinical, imaging, and pathophysiological information for the 26 patients, including ours, in 12 studies [1,2,4,5,6,7,8,9,10,11,12] were collected and analyzed systemically. Time to recovery was defined by the description that a patient was able to stand or walk independently or with minimal assistance. A partial recovery was defined as the patient needing assistance to stand or walk. The IBM^®^ SPSS^®^ Statistics version 20.0 was used for statistical analysis. Independent Student’s *t*-test was used for scale variables. A statistical significance was defined as a *p* value of <0.05.

## 3. Results

Table 1 summarizes all non-psychogenic astasia patients reported in the English literature from 1976 to 2019. The demographic data, associated symptoms, mechanism, and laterality of astasia, proposed affected pathway, and the recovery period of astasia were shown. A total of 26 patients, including ours, were categorized in order of location of lesions from the brainstem, thalamus, cerebral white matter, and cortex. The intention was to provide a virtual illustration of the involved projection fibers responsible for astasia. 

In these 26 patients, there were 19 men and 7 women with a mean age of 67.8 years. Nineteen (73%) reported cases, all located at the brainstem or thalamus, had associated symptoms, including gaze palsy, asterixis, and sensory deficits. Specifically, the characteristics of laterality of pulsion movement were reviewed, and most cases reported contralateral lateropulsios or retropulsions. Infarction, hemorrhage, and tumor were three causes reported in non-psycogenic astasia, fourteen (54%) were due to infarctions, nine (35%) were due to hemorrhages and three (11%) were due to tumors. For the location of lesions, three patients (11%) had lesion in the brainstem, seventeen patients (65%) had lesion in the thalamus, two patients (8%) had lesion in the cerebral white matter, and four patients (15%) had lesion in the cerebral cortex. Sixteen patients (62%) had their lesions on the right side, nine patients (35%) on the left side, and one patient had a midline lesion (our case). For the affected pathway, a comprehensive discussion is made in the discussion paragraph. Despite various causes, only four cases did not recover well; three were caused by tumors (two in Masdeu’s series [5] and our case), while twenty-two cases (85%) were considered as recovered (patients were able to stand or walk independently or with minimal assistance) with a mean duration of 16 days.

Table 2 analyzes the time to recovery from astasia for different causes. A trend towards a recovery from infarction sooner than from hemorrhage was found, with a mean time to recovery of 10.6 days compared to that of 36.9 days for hemorrhagic causes (*p* = 0.058). For the two patients with thalamic tumors reported by Masdeu [1], however, did not receive treatment and astasia progressed without recovery.

Table 3 analyses the time to recovery from astasia by different locations. The average time to recovery was 61.6 days in the brainstem group, 14.9 days in the thalamus group, 92 days in the cerebral white matter group, and 16.5 days in the cortex group. A significant longer time to recovery was observed when the lesion was in the brainstem compared to the thalamus (*p* = 0.007), while no difference was found when comparing lesions in the thalamus to lesions in the cortex (*p* = 0.306).

In summary, we summarized and analyzed all 26 non-psychogenic astasia patients that were in the English literature. Seventy-three percent of them were men (n = 19), seventy-three percent were associated with other neurologic symptoms (n = 19) and sixty-two percent of the reported lesions were on the right side (n = 16). Contralateral lateropulsion was very common, followed by retropulsion, when describing astasia. Infarctions were the most reported cause (n = 14, 54%). The thalamus was the most reported location (n = 17, 65%). An association between the cause of astasia and time to recovery was found, with infarctions being the most likely to recover with a mean of 10.6 days. Lesions at the brainstem had a longer time to recovery with a mean of 61.6 days.

## 4. Discussion

### 4.1. Graviceptive Systems: Vestibule-Thalamic Projections

The link between astasia and the vestibule-thalamic pathways clearly declares that the associated network is in the vestibular system, which has a unique role in sensorimotor control and perception [20]. Multiple thalamic nuclei are incorporated in the vestibular processing, including the ventroposterior complex, the ventroanterior–ventrolateral complex, the intralaminar nuclei, and the posterior nuclear group (medial and lateral geniculate nuclei, pulvinar) [20]. Specifically, posterolateral thalamic lesions may present a tilt of the perceived visual vertical [21,22] and postural instability [23,24]. Therefore, by relaying vestibular cues, particularly graviceptive cues, this thalamic region could be involved in encoding gravity and controlling body orientation in space [20]. Subjective visual vertical (SVV) tilts are the most sensitive sign of a vestibular tone imbalance in roll and occur with peripheral or central vestibular lesions from the labyrinthine to the vestibular cortex [21]. Two graviceptive systems have been introduced. The primary graviceptive system is the perception of the visual world and is characterized by an SVV tilt either ipsiversively (ipsilateral eye undermost) or contraversively (contralateral eye undermost) [21]. The two neural pathways that were identified are responsible for these tilt symptoms, (1) a crossed graviceptive pathway involving the medial longitudinal fasciculus to the interstitial nucleus of Cajal may result in ipsi- or contraversive tilt depending on the location of the lesion [21]; and (2) an ipsilateral graviceptive pathway running from vestibular nuclei close to and within the medial lemniscus to the posterolateral thalamus results in only ipsiversive tilt [25].

The second graviceptive system proposed by Karnath et al. is located in the posterior thalamus; therefore, we argue that this area is not only a “first-order relay structure” of the vestibular pathway [24,26], but a “higher order” relay station [27]. They have described a symptom named “contraversive pushing” or “pusher syndrome” that is presented in patients with thalamic stroke. When at rest and also when asked to sit up, pusher patients extend the unaffected arm and use it to push away actively from the nonparetic side [26]. Moreover, pusher patients experience their body as oriented “upright” when the subjective postural vertical (SPV) is tilted 18° to the nonhemiparetic, ipsilesional side. In contrast, the perception of the SVV was undisturbed [24]. Both pusher syndrome and astasia are disturbances in the control of upright body posture and involve the posterior thalamus. Nevertheless, they seem to be different in several aspects: First, astasia patients have minimal or no weakness in muscle power, while pusher patients are hemiparetic. Second, astasia patients present with contralateral lateropulsion, which fell “without” a push from either side of the extremity, while pusher patients had their nonparetic arm push away “actively” from the nonparetic side (fell contralaterally). Third, and probably the most distinctive one, is that astasia patients perceive a tilted SVV [21,22,28,29], while pusher patients perceive a severe ipsiversive tilted SPV with SVV undisturbed [7,23,24,26]. However, one may argue that no SPV data have been reported in astasia patients. In addition, it has been demonstrated that the possible cortical network of the second graviceptive system includes the inferior frontal gyrus, middle temporal gyrus, inferior parietal lobule, and parietal white matter, by showing hypoperfusion in these regions [30]. This pattern of cortical areas provides indistinguishable information from the primary graviceptive system, which was primarily considered a project of the parietal-insular vestibular cortex (PIVC) [21]. Although anatomically overlapped, a functional discrepancy has already been suggested that the perceived relation of the visual world to the vertical is exclusively determined by sense organs in the head, whereas body posture is also directly measured by the recently discovered graviceptors in the human trunk [31].

We now inspect the phenomenon of body lateropulsion. Since this is presented in nearly all reported patients with astasia, body lateropulsion may be considered a physical biomarker for astasia (Table 1). From a review by Dieterich et al., it has been shown that lesions involving a caudal medullary lesion of the spinocerebellar tract, the descending lateral vestibulospinal tract, the ascending vestibulo-thalamic and dentatorubro-thalamic pathways, or the thalamocortical fascicle were associated with lateropulsions [29]. The majority of reported cases with isolated symptomatology of lateropulsion were attributed to lesions in the brainstem, e.g., Wallenberg syndrome [15,29]; despite the rarity, we have added cases from different anatomical locations with body lateropulsion in this review. In our opinion, the evidence seemed to guide us to the primary graviceptive system as the responsible pathways, including ipsilateral vestibulothalamic pathway and crossed vestibulothalamic pathway, for astasia. Afterwards, two anatomically distinct graviceptive signal processing systems within the thalamus, which resulted in contra- or ipsi-versive tilt of SVV, have been elucidated [32].

### 4.2. Thalamo-Cortical Projections: Subcortical White Matters

A wide and extensive network interconnection has been delineated between the thalamus and multiple “vestibular cortex” areas in the primary graviceptive pathways [33,34]. Not two but five separate and distinct vestibular pathways were identified thereafter: three run ipsilaterally, while the two others cross either in the pons or the midbrain. Two of the ipsilateral projections run through the posterolateral or paramedian thalamic subnuclei, while the third bypasses the thalamus to reach directly the inferior part of the insular cortex. Both contralateral pathways travel through the posterolateral thalamus [34]. At the cortical level, the PIVC regions of both hemispheres with a right hemispherical dominance are interconnected transcallosally through the antero-caudal splenium [34]. In our case and one case presented by Zhang et al. [10], the corpus callosum has been involved. However, it was our case that involved the whole body of CC from genu to splenium. We believe this explains why in our patient astasia could improve partially, owing to a significantly reduced mass effect on the subcortical white matter pathway bilaterally after surgery and adjuvant therapies but never fully recover, owing to a loss of connectivity between the bilateral hemispheric vestibular pathways. We also believe, aside from the bilaterally projected fastigial outputs, a bi-hemispheric compensation of vestibular function via transcallosal communication contributed to a rapid recovery of a balanced upright posture in both infarction and hemorrhage-related astasia patients found in the literature.

### 4.3. Thalamo-Cortical Projections: Cortical Level

**Figure 3:**
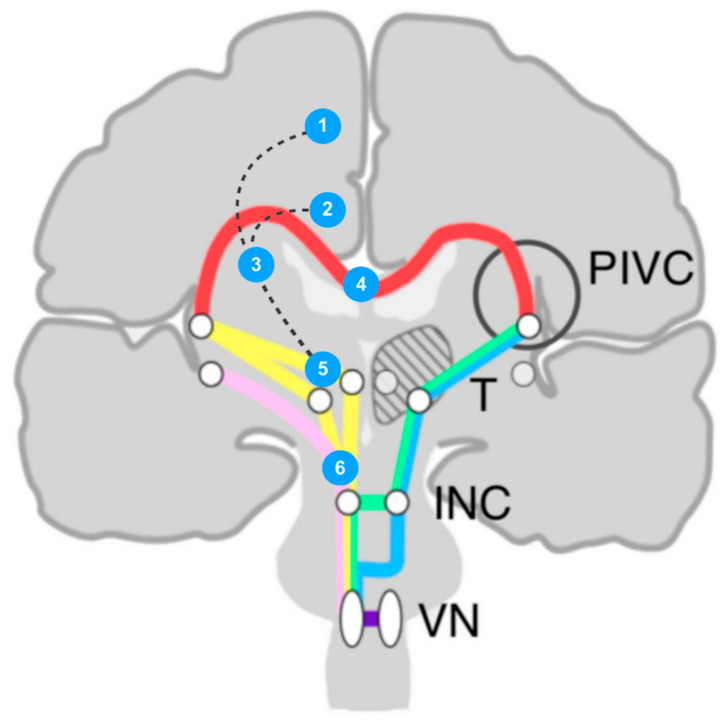
An illustration of cases with astasia in the English literature on the schema of vestibular rope ladder system. To simplify, all 26 cases are presented by numbers at different anatomical structures and are shown ipsilaterally. 1: supplementary motor area (n = 1); 2: cingulate gyrus (n = 3); 3: suprathalamic white matter (n = 1); 4: corpus callosum (n = 1); 5: thalamus (n = 17); 6: mesencephalon (n = 3). The rope ladder system demonstrates the basic network of vestibular system. PIVC: parietal insular vestibular cortex; T: thalamus; INC: interstitial nucleus of Cajal; VN: vestibular nuclei. (adapted and modified from Kirsch et al., 2016 [34], with permission).

It has been summarized that apart from PIVC, there were vestibular responses recorded in the somatosensory cortex, intraparietal sulcus, posterior parietal cortex, medial superior temporal area, frontal cortex (primary motor and SMA), cingulum, and hippocampus [20]. Cingulate motor areas that enfold the cingulate sulcus have been studied primarily in nonhuman primates [35,36]; even so, human evidence has been provided by a real-time motor representation using subdural electrode recording [37]. A significant corticocortical interconnection between SMA, frontal eye field, motor cortex, and CMAs represent its function in movement initiation and modification [35,36,37,38]. Therefore, the cingulum, not only CMAs, has abundant connectivities which are subtended by pathways running from the thalamus as well as the intercortical areas. All in all, the vestibular system, composed of the graviceptive system (vestibule-thalamic pathways) and thalamo-cortical projections, adequately describes all reported astasia patients in the literature. Interestingly, all reported cortical regions were along the medial hemispheric surface, such as the cingulate gyrus and SMA (Table 1 and Figure 3). Whether the regional preference has its biological significance or not remains to be determined.

There are several limitations to this study. First, publication bias and selection bias are inevitable, since we selected only reports that used “astasia” in the topic from the English literature. On the other hand, we may only understand better the pathophysiology of astasia through these selected representative cases. Second, we searched PubMed and Google Scholar but no other databases, such as SCOPUS, Web of Science, PEDro, or EMBASE. Third, one should be aware that lesions affecting pathways associated with astasia might be more common than reported; however, more damage resulting in serious symptoms may mask astasia from clinical detection. Fourth, this being a retrospective review, we could only provide a correlation analysis between a known deficit and proposed pathways. For such a rare symptom, despite the difficulties, a larger patient cohort or delicate imaging examinations may be needed to validate our findings.

## 5. Conclusions

In conclusion, we summarize that astasia always presents with lateropulsion of the body, and the underlying interrupted pathway may be the primary graviceptive system, which is composed of at least five unilateral and contralateral projection fibers from vestibular nuclei to thalamic nuclei and thalamo-cortical projections including subcortical white matter tracts and cortical areas. Future studies on astasia should include SVV and SPV to elucidate the pathological changes in the perception of verticality in the visual world and the body. A symptomatology or electrical stimulation comparative study on controlling body posture on the medial hemispheric versus lateral hemispheric vestibular cortices may help to understand the differences between these areas.

## Figures and Tables

**Table 1:** Summary of all reported nonpsychogenic astasia-abasia patients in the English literature from 1976–2019 (n = 26).

Author	Case Num.	Age (yr)/Sex	Associated Symptoms †	Laterality of Pulsion	Causes	Location	Laterality of Lesion	Proposed Affected Pathway ^$^	Recovery of Astasia (Days) ^¶^
Masdeu, et al. [5]	1	83/M	Up-gaze palsy	Retropulsion	Hemorrhage	Pontomesencephalic junction (DM)	R	Vestibulocerebellar pathway	>120 (partial)
Song, et al. [6]	1	70/M	Asterixis	CL lateropulsion or retropulsion	Infarction	Medial thalamo-mesencephalic	L	Vestibulocerebellar pathway	5
Pablo-Fernandez, et al. [4]	1	58/M	Gaze palsy, asterixis	CL lateropulsion or retropulsion	Hemorrhage	Medial thalamo-mesencephalic	R	Vestibulocerebellar pathway	60
Masdeu, et al. [5] ^§^	7	65.4/6 M, 1 F	Sensory deficits	CL lateropulsion or retropulsion	Hemorrhage	Thalamus (dorsal *)	4 R, 3 L	Vestibulocerebellar pathway	21.7 (mean)
	6	73.6/3 M, 3 F	Sensory deficits		Infarction	Thalamus (VL ^‡^)	4 R, 2 L		4.6 (mean)
	2	60.5/1 M, 1 F	Sensory deficits		Tumor	Thalamus (AL, VB)	2 R		Progressive
Lee, et al. [8]	1	76/F	Sensory deficit	Antepulsion or retropulsion	Infarction	Thalamus (PL)	R	Second graviceptive pathway	2
Elwischger, et al. [7]	1	82/M	None	CL lateropulsion	Infarction	Thalamus (CM)	L	Crossed graviceptive pathway	42
Takahashi, et al. [9]	1	73/F	None	Retropulsion	Infarction	Suprathalamic white matter	R	Thalamo-cortical projection	4
Chen, et al. (present case)	1	50/M	None	Retropulsion or either sides	Tumor	Corpus callosum body	Bil	Thalamo-cortical pathway, bil	>180 (partial)
Zhang, et al. [10]	1	65/M	None	CL lateropulsion, retropulsion	Infarction	Anterior cingulate, corpus callosum	R	Vestibulocerebellar pathway; disconnection	30
Kataoka, et al. [11]	1	67/M	None	CL lateropulsion	Infraction	Posterior cingulate	L	Cingulate motor area	14
Satow, et al. [2]	1	58/M	None	CL lateropulsion	Infarction	Middle cingulate	L	Cingulate motor area	21
Wada, et al. [12]	1	61/M	None	CL lateropulsion	Infarction	Supplementary motor area	R	Vestibulocerebellar pathway	2
Summary	26	67.8/19M, 7F	19 with and 7 without associated symptoms	CL lateropulsion and retropulsion	Infarction (14), hemorrhage (9), tumor (3)	Brainstem (3), thalamus (17), White matter (2), cortex (4)	R (16), L (9), Bil (1)	Primary graviceptive system and thalamo-cortical projections *See “Discussion”*	32.7 (mean) Total: 16.4 Partial: >160

AL: anterolateral; Bil: bilateral; CL: contralateral; CM: centromedian; DM: dorsomedial; F: female; L: left; M: male; PL: posteriolateral; R: right; VB: ventrobasal; VL: ventrolateral. † Associated symptoms were defined as neurological deficits described in the study. ^$^ All affected pathways proposed in each study were recorded, and a summary was made after a detailed discussion and literature review. ^¶^ Time to recovery was defined by the description that a patient was able to stand or walk independently or with minimal assistance. A partial recovery was defined as the patient need assistance to stand or walk. * According to Masdeu et al., dorsal*4, dorsoposteriorlateral*1, dorsomedial*1, and lateral*1. ^‡^ According to Masdeu et al., ventrolateral*4, suprathalamic*1 and only anteriomedial spared*1. ^§^ Age and recovery period were presented as mean.

**Table 2:** Analysis of time to recovery of astasia by causes (n = 26).

Cause	Case Number	Age (Years, Mean)	Location	Time to Recovery ^¶^ (Days, Mean ± SD)
Infarction	14	71	Brainstem (1) Thalamus (8) White matter (1) Cortex (4)	10.6 ± 12.3
Hemorrhage	9	66.6	Brainstem (2) Thalamus (7)	36.9 ± 35.1
Tumor	3	57	Thalamus (2) White matter (1)	>180 *

SD: standard deviation. ^¶^ A Student’s *t*-test showed a trend toward shorter time to recovery in infarction group than in hemorrhage group (*p* = 0.058). * Two of three cases did not report the outcome of astasia. SD: standard deviation.

**Table 3:** Analysis of time to recovery from astasia by lesion locations (n = 26).

Location	Case Number	Age (Years, Mean)	Cause	Time to Recovery ^¶^ (Days, Mean ± SD)
Brainstem	3	70.3	Hemorrhage (2), Infarction (1)	>61.6 ± 57.5
Thalamus	17	69.4	Hemorrhage (7), Infarction (8), Tumor (2)	14.9 ± 13.9 *
Cerebral whit matter	2	61.5	Infarction (1) Tumor (1)	>92
Cortex	4	62.8	Infarction (4)	16.5 ± 11.9

SD: standard deviation. ^¶^ A Student’s *t*-test showed a statistically and significantly longer time to recovery when the lesion was in the brainstem than in the thalamus (*p* = 0.007); while no difference was found when comparing lesions in the thalamus to lesions in the cortex (*p* = 0.306). * Two cases caused by tumors were excluded from the time to recovery analysis, since these two patients progressed rather than recovered.

## Data Availability

Not applicable.

## Abbreviation

CC: corpus callosum; CMA: cingulate motor area; IDH: isocitrate dehydrogenase; MRI: magnetic resonance imaging; PIVC: parietal insular vestibular cortex; SMA: supplementary motor area; SPV: subjective postural vertical; SVV: subjective visual vertical; T2-WI: T2-weighted imaging; WHO: World Health Organization.
